# IL-3 and Oncogenic Abl Regulate the Myeloblast Transcriptome by Altering mRNA Stability

**DOI:** 10.1371/journal.pone.0007469

**Published:** 2009-10-15

**Authors:** Jason Ernst, Louis Ghanem, Ziv Bar-Joseph, Michael McNamara, Jason Brown, Richard A. Steinman

**Affiliations:** 1 Computer Science and Artificial Intelligence Laboratory, Massachusetts Institute of Technology, Boston, Massachusetts, United States of America; 2 Department of Pediatrics, Children's Hospital of Philadelphia, Philadelphia, Pennsylvania, United States of America; 3 Department of Computer Science, Carnegie Mellon University, Pittsburgh, Pennsylvania, United States of America; 4 Regional Oncology Department, The Cleveland Clinic, Cleveland, Ohio, United States of America; 5 Oncology staff, Meadeville Medical Center, Meadevill, Pennsylvania, United States of America; 6 Department of Medicine, University of Pittsburgh School of Medicine, Pittsburgh, Pennsylvania, United States of America; Roswell Park Cancer Institute, United States of America

## Abstract

The growth factor interleukin-3 (IL-3) promotes the survival and growth of multipotent hematopoietic progenitors and stimulates myelopoiesis. It has also been reported to oppose terminal granulopoiesis and to support leukemic cell growth through autocrine or paracrine mechanisms. The degree to which IL-3 acts at the posttranscriptional level is largely unknown. We have conducted global mRNA decay profiling and bioinformatic analyses in 32Dcl3 myeloblasts indicating that IL-3 caused immediate early stabilization of hundreds of transcripts in pathways relevant to myeloblast function. Stabilized transcripts were enriched for AU-Response elements (AREs), and an ARE-containing domain from the interleukin-6 (IL-6) 3′-UTR rendered a heterologous gene responsive to IL-3-mediated transcript stabilization. Many IL-3-stabilized transcripts had been associated with leukemic transformation. Deregulated Abl kinase shared with IL-3 the ability to delay turnover of transcripts involved in proliferation or differentiation blockade, relying, in part, on signaling through the Mek/Erk pathway. These findings support a model of IL-3 action through mRNA stability control and suggest that aberrant stabilization of an mRNA network linked to IL-3 contributes to leukemic cell growth.

## Introduction

The growth factor Interleukin-3 (IL-3) is a pleiotropic cytokine protein that promotes the survival and proliferation of multipotent hematopoietic progenitors, and stimulates the development of multiple hematopoietic lineages. While IL-3 supports early myelopoiesis, it opposes terminal granulocytic differentiation. We and others have shown that IL-3 prevents execution of the G-CSF differentiation program in the murine 32Dcl3 model of granulopoiesis [1]–[3]; IL-3 also opposes terminal granulocytic differentiation of normal human myeloid precursor cells [4], [5].

Heightened expression of hematopoietic growth factors such as IL-3 could support leukemia progression through an autocrine mechanism. High expression of the IL-3 receptor has been noted in leukemic blasts [6] and correlates with enhanced cycling of blasts and poor patient oucomes [7]. IL-3 is frequently expressed by leukemic myeloblasts [8] and can promote clonogenicity of primary blasts [9]. IL-3 production by imatinib resistant CML blasts can render other leukemic blast cells resistant through paracrine stimulation [10]. In one experimental leukemic model, downregulation of an IL-3 autocrine loop decreased leukemogenicity [11].

While most investigations of the action of hematopoietic cytokines such as IL-3 have focused on cytokine signal transduction leading to transcriptional activation (reviewed in [12]), limited evidence suggests that the cellular response to IL-3 cytokine stimulation also involves posttranscriptional mechanisms. Matsui et. al. [13] have reported that Bim-mediated apoptosis is prevented in an IL-3- dependent B-cell line by IL-3-mediated decay of Bim transcript. In addition, IL-3 was shown to increase mRNA stability of its own receptor subunit in mature eosinophils [14]. We sought to determine whether proliferating myeloblasts expressed a distinct profile of mRNA half-lives that was controlled by hematopoietic cytokines such as IL-3.

The regulation of mRNA turnover often occurs via the 3′-untranslated region (3′-UTR) at AU-rich elements (ARE's) that are recognized and competitively bound by RNA-binding proteins that stabilize or destabilize the transcripts. Roughly 4000 genes are known to contain these elements and consist primarily of short-lived transcripts. Three classes of AU-rich sequences have been described including those with single or noncontinuous canonical UUAUUUAWW motifs (Class I), those containing overlapping or continuous copies of the motif (Class II), and a 3^rd^ class of U-rich sequences lacking that the AU-motif (Class III) [15]–[17].

Coordinate stabilization of multiple mRNAs allows coherent cellular responses to external stimuli. Several studies indicate that mRNAs in the same pathways share similar decay kinetics and could share overlapping stability control pathways [18]. For instance, interferon gamma synchronously increases expression of proinflammatory molecules via mRNA stabilization [19]. The coordinate stabilization of multiple transcripts has also been reported upon T-cell activation [20] and during muscle and neuronal differentiation [21], [22]. However, no systematic analysis of mRNA turnover in myeloblasts exposed to proliferative hematopoietic cytokines has been performed. Better understanding of the control of mRNA metabolism in myeloblasts could illuminate posttranscriptional defects that occur in myeloproliferative disorders or myeloid leukemias.

As a model for studying hematopoietic cytokine regulation of mRNA turnover, we have used the murine myeloblast cell line 32Dcl3. These nonleukemic, diploid cells are IL-3-dependent for proliferation and survival and differentiate into functional neutrophils when IL-3 is replaced by G-CSF in culture [23]. 32Dcl3 cells have frequently been used to analyze molecular components of cytokine signaling pathways [24]–[26].

We have previously reported that 32Dcl3 myeloblasts growing in IL-3 express high basal levels of p21WAF1 mRNA and protein, and that p21 inhibits the neutrophilic differentiation of these cells [27]. Because reports in nonhematopoietic cells have demonstrated that p21 transcript turnover changed in response to cellular stress, growth factors and differentiation stimuli, we posited that IL-3 induction of p21 could reflect mRNA stabilization. In this report, we demonstrate that p21 is just one of many transcripts that are stabilized by IL-3. Of note, IL-3 stabilized these mRNAs without the need for IL-3- mediated transcription because IL-3 was added after transcriptional blockade. These posttranscriptionally regulated mRNAs are functionally coordinated, mapping to several key pathways characteristic of the myeloblast phenotype. In particular, IL-3 opposed the decay of transcripts related to cell cycling. Bioinformatic analysis indicated enrichment of AU-rich sequences in the 3′ UTR of transcripts stabilized by IL-3. These findings support a model in which IL-3 supports myeloblast growth through signals that encroach on shared 3′-UTR motifs in a substantial portion of the transcriptome.

Oncoproteins such as v-Abl can render 32D myeloblasts IL-3-independent for growth. Activated Abl kinase has been shown to overlap with IL-3 in its activation of downstream signals [28]. We sought to determine whether v-Abl kinase activity could mimick IL-3 in controlling the rate of decay of transcripts linked to myeloblast growth and survival. Results indicated that Abl kinase slowed the decay of select transcripts, similar to IL-3.

## Results

### p21 as a model for posttranscriptional control by IL-3

**Figure 1:**
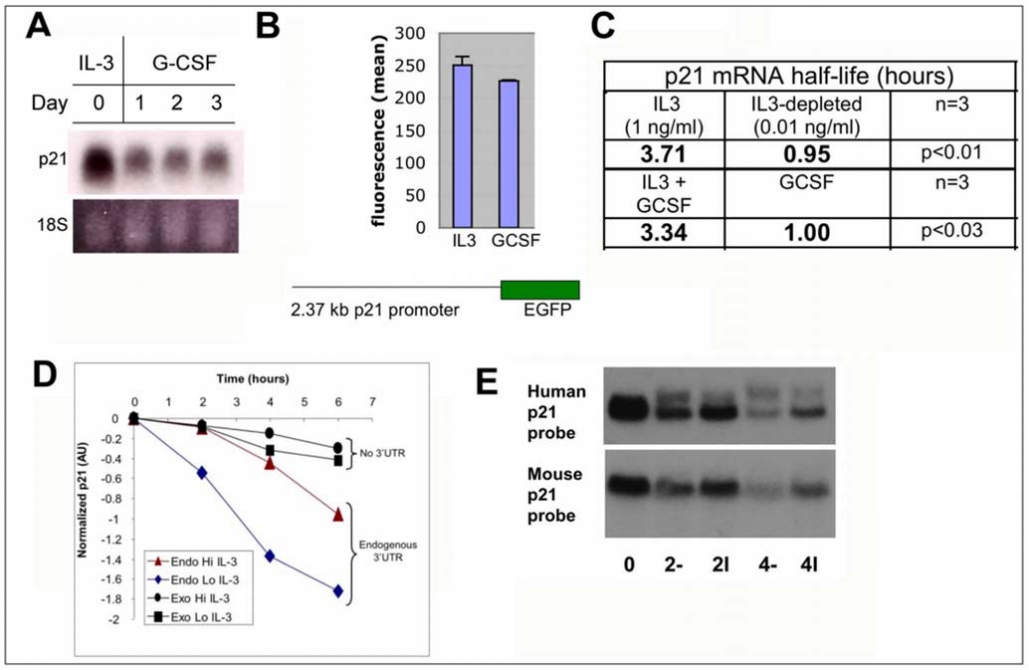
Posttranscriptional p21 mRNA regulation by IL-3. A) *Downregulation of p21 mRNA in differentiating myeloblasts*. Northern blot analysis of total RNA from 32Dcl3 cells propagating in IL-3 (1 ng/mL) or following treatment with G-CSF (100 ng/mL) for indicated times. Cells remained viable over this time course. RNA harvested daily was probed with ^32^P-labeled murine p21 cDNA. Ethidium bromide stained 18S RNA is provided as a loading control. B) *Comparable p21 promoter activity in proliferating and differentiating myeloblasts*. 32Dcl3 cells stably transfected with EGFP driven by the murine p21 promoter were washed and either resuspended in IL-3 containing medium (1 ng/ml) or in G-CSF-containing medium (30 ng/ml) and EGFP expression measured 48 hours later by flow cytometry. Experiment was done twice with triplicate determinations. C) *IL-3 stabilizes p21 mRNA*. The half-life of p21 under indicated conditions was determined as described in Materials and Methods. p21 signals normalized against GAPDH on autoradiograms were plotted and mean decay rate of 3 independent experiments were determined. GAPDH remains stable under these conditions (Supplemental Table S1). D) *IL-3-regulated turnover requires 3-UTR*. Cells were stably transfected with p21 lacking UTR sequences, and decay of endogenous p21 (endo) compared to exogenous p21 (exo) cDNA was measured in chase experiments as above. E) *Comparable decay of human and murine p21*. Stable transfectants containing human p21 mRNA including the full-length 3′-UTR were exposed to medium with (+) or without (−) IL-3 for 2 or 4 hours after Actinomycin D as indicated, and mRNA blotted.

In the 32Dcl3 myeloblast model, IL-3 supports proliferation and survival and inhibits cellular differentiation. If IL-3 coordinates this phenotype in part through stabilizing mRNAs, then a subset of transcripts linked to these functions will decay slower in IL-3 than when IL-3 is withdrawn or replaced with the differentiating cytokine G-CSF. Figure 1 demonstrates that p21 mRNA levels decrease when cells are switched from IL-3 to G-CSF (Figure 1A). In order to determine whether cytokine exposure directly affected transcription of p21, 32Dcl3 cells were stably transfected with an EGFP reporter driven by the p21 promoter. Cell were washed, resuspended in IL-3 or G-CSF, and EGFP fluorescence was measured two days later using flow cytometry. EGFP expression varied by less than 10% between culture conditions, indicating a minimal effect of IL-3 on p21 transcription and bolstering a posttranscriptional basis for differences in p21 mRNA levels (Figure 1B). Actinomycin D chase experiments confirmed that the half-life of p21 in physiologic [29] (1 ng/ml) IL-3 was roughly 3.5 times longer than when cells were maintained in IL-3 –deficient (0.01 ng/ml) medium or in G-CSF (Figure 1C). Furthermore, the addition of IL-3 to cells cultured overnight in G-CSF restored p21 stability. Stably transfected 32Dcl3 cells harboring exogenous human p21 cDNA lacking the 3′-UTR (untranslated region) exhibited increased stability that did not change significantly in IL-3-deficient medium. In contrast, the mRNA signal corresponding to endogenous p21 bearing the full-length 3′-UTR was markedly less stable in IL-3-deficient medium than with physiologic IL-3 (Figure 1D). Having shown that IL-3 upregulated p21 mRNA by increasing it's stability in 32Dcl3 mouse myeloblasts, we determined whether human p21 was also subject to similar regulation. Full-length human p21 (including untranslated regions) cDNA was stably transfected into 32Dcl3 cells. The decay of exogenous human p21 and endogenous mouse p21 transcripts were detected using species-specific probes on Northern blots of Actinomycin D chase experiments (1E). IL-3 stabilized transcripts of either species similarly. This indicated that enhanced expression of p21 in 32Dcl3 myeloblasts reflected a longer p21 mRNA half-life due to IL-3 that was dependent on the 3′-UTR of the p21 transcript.

### IL-3 stabilizes a network of transcripts involved in cell cycling

We have previously demonstrated that specific RNAi-mediated downmodulation of p21 accelerated granulocytic differentiation in the 32Dcl3 cell line [27], indicating a differentiation-suppressive role for p21 in these myeloblasts. The posttranscriptional stabilization of p21 by IL-3 (noted above) could contribute to IL-3 blockade of 32Dcl3 cell differentiation. We therefore determined whether IL-3 altered the turnover of a number of transcripts linked to myeloblast growth or function, in addition to p21. In order to derive a global list of candidate IL-3 targets, we conducted kinetic microarrays [30] in which 32Dcl3 cells were washed free of IL-3, exposed to actinomycin D to stop transcription and then treated with either IL-3-replete or IL-3-deficient medium (Supplemental Figure S1). Cells were harvested 2 and 4 hours after cytokine re-addition and Affymetrix 430A-2.0 microarrays were run on samples harvested from each condition at 0, 2 and 4 hours. These time points were chosen to capture labile, cytokine-responsive transcripts within a temporal window in which cells were fully viable. Two independent experiments were run. A series of analyses were conducted in order to rank-order probe sets by the degree to which IL-3 stabilized the corresponding transcripts (see Supplemental Methods S1).

To perform these analyses, a novel algorithm was developed. The degree to which IL-3 stabilized a transcript was denoted by the difference between the transcript's decay rate in cells exposed to IL-3 replete- versus IL-3-deficient medium. The algorithm begins by calculating the average area between the mRNA decay curve of each gene probe set in the presence of IL-3 and the decay curve of that probe set in IL-3-deficient medium (*g_area_*). The greater the value of *g_area_*, the larger the difference in transcript decay with and without IL-3 depletion. We then adjusted *g_area_* based on the time point 0 signal intensity to correct for the greater effect that noise would have on *g_area_* for probes with the lowest signal intensity (see Supplemental Methods S1). This adjustment led to a score for each probe set termed *g_score_*. Higher *g_score_* values were expected to correspond to increased transcript stabilization by IL-3 as manifested by a greater difference in the adjusted area between the decay curve in IL-3-replete and -deficient medium. We used our algorithm to rank order the 22,690 probe sets from those corresponding to transcripts that were the most-stabilized by IL-3 to those least-stabilized by IL-3 (See Supplemental Methods S1), as shown on scatter plots (Supplemental Figure S2).

Confidence in the rank-ordered list was bolstered by the identification of p21 as a strongly stabilized transcript. Out of the 22,690 probe sets on the microarray, the two probes corresponding to p21 occupied the 14^th^-most and 17^th^-most stabilized positions. The probesets included on microarrays used for these studies represent 13,486 unique transcripts based on Entrez gene ID's. When ordered by unique transcript (i.e. averaging out probesets that recognize distinct portions of the transcript), p21 was the 10^th^-most stabilized transcript out of 13,486. The list of probesets arranged by IL-3-mediated stability is shown in Supplemental Table S1. A table of genes ranked from most- to least-stablized derived from taking the mean value for probesets annotated to the same gene is shown in Supplemental Table S2. The listings in both supplemental tables were in close agreement in the sense that we found either list would produce similar results in our downstream analysis.

The primary conclusion from this microarray analysis was that transcripts represented by 5290 probe sets were measured to decay slower in 1 ng/ml IL-3 than without it, with most of the total measured magnitude of stabilization occuring in the top 1000 probe sets (Table S1, Figure S2). IL-3 was therefore concluded to stabilize hundreds of gene transcripts in 32Dcl3 myeloblasts.

**Figure 2:**
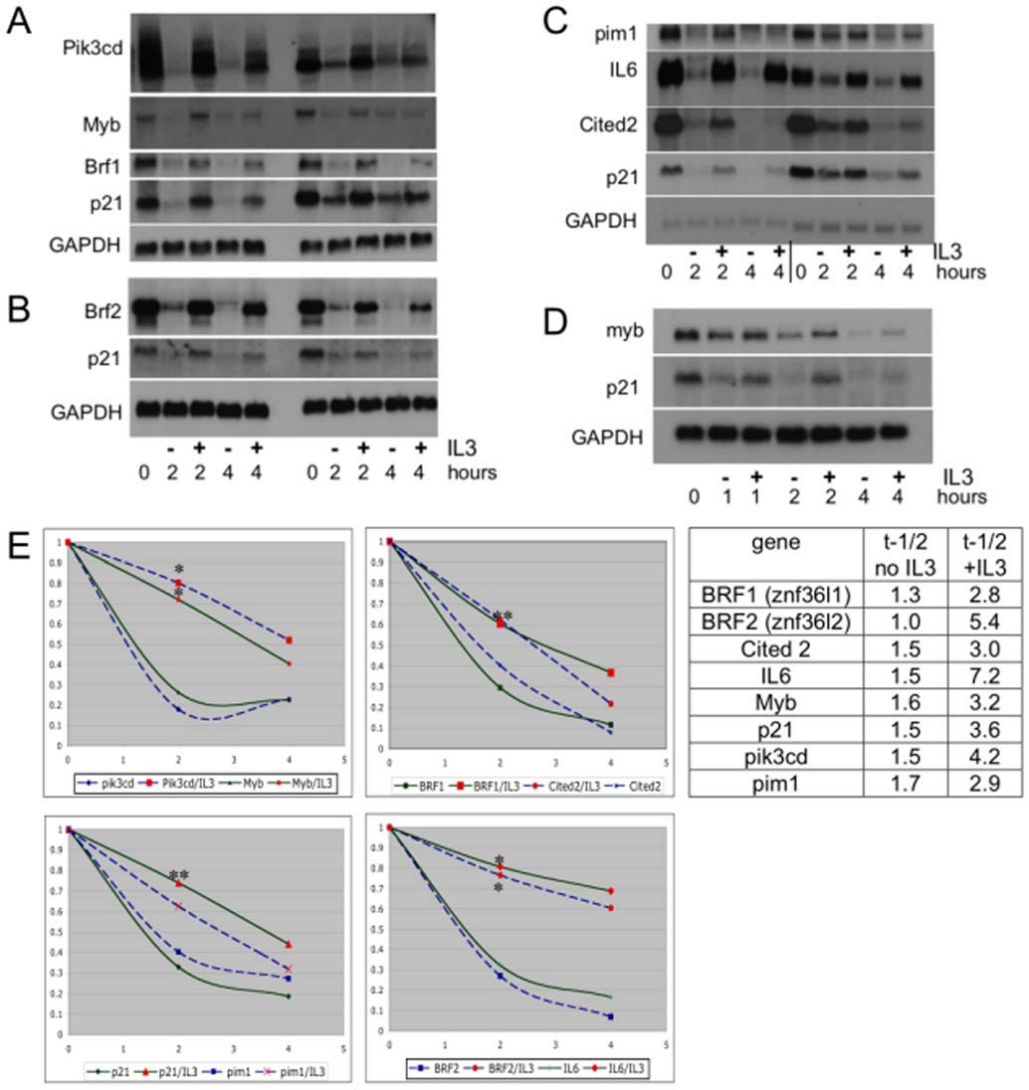
IL-3 regulated turnover of transcripts. *A–D. Northern Blots of IL-3 stabilized transcripts*. Northern blots of samples from one (*D)* or two independent experiments each (*A, B, C)*. RNAs were prepared from 32Dcl3 cells washed free of IL-3, transcriptionally arrested and then exposed to medium alone (−) or reexposed to 1 ng/ml IL-3 (+) for indicated times. Blots were probed with riboprobes prepared for transcripts selected from microarray-based rank listings. GAPDH expression is shown as a loading control. p21 is probed as a positive control in each of the 7 experiments. For each time point, lower expression of the sample lacking IL-3 (−) is evident. *E. left: Graphic representation of Northern blot results*. Decay curves are plotted from the data in *A–D* quantitated with Image J software. *p< = 0.05, **p<0.002 comparison of value with and without IL3. *right*: Transcript half-lives calculated as described in Methods for IL-3 positive and -negative conditions.

In order to validate microarray-based rankings, Northern blotting for p21 and several other transcripts was conducted on extracts of cells from Actinomycin D chase experiments conducted in the presence or absence of IL-3 (Figure 2A). For each of seven independent experiments shown on Northern Blots, p21 was probed as a positive control and manifested a decay half-life of 3.6 hours in IL-3 and 1.5 hours without IL-3 on average (Figure 2B). The probe sets for the loading control utilized, GAPDH, were at positions 10,018, 10,420, and 14,173 out of all queried probe sets and manifested minimal cytokine-dependent variation. It was evident in each case that IL-3 dramatically retarded the degradation of transcripts that otherwise occurs in the absence of this cytokine. In several cases transcripts were undetectable within 2 hours unless IL-3 was present. Results are plotted in Figure 2B. It is notable that the IL-3 stabilized transcripts shown on duplicate Northern Blots in Figure 2 have been associated with regulatory roles in normal or malignant hematopoiesis (Table 1).

**Table 1:** Hematopoietic roles of validated IL-3 posttranscriptional targets.

Gene name	position in rank list	Notes	reference
Pik3cd (PI3-kinase delta)	3	•associated with myeloid leukemia	[52], [53].
		•supports leukemic cell growth and survival	
IL-6	5	• affects differentiation or proliferation	[54]
		•produced by AML blasts	
zfp36l1 (Brf1, tis11b)	13	•associated with t(8∶11) myeloid leukemia proliferation	[55]
cdkn1a (p21)	14	•inhibits 32D differentiation	[27], [56]
		•associated with resistant AML	
zfp36l2 (Brf2, tis11d)	39	•upregulated in resistant myeloid leukemia	[57], [58]
		•involvement at a leukemic breakpoint	
myb	58	•role in hematopoiesis and promotion of leukemia	[59]–[61]
		•inhibits 32Dcl3 differentiation and apoptosis	
pim-1	103	•role in Flt3-mediated survival in myeloid leukemia	[62]
cited-2	273	•essential for hematopoiesis	[63]

The ranked position (out of 22,690 probe sets assayed) of Northern-blot-validated transcript probe sets and references highlighting their role in normal or malignant hematopoiesis are shown. For transcripts linked to multiple probe sets, the first occurrence of transcript in the ranked probe set list is shown.

### Functional grouping of transcripts stabilized by IL-3

In order to uncover shared biological functions between the transcripts that were stabilized by IL-3, gene ontology (GO) analysis was conducted for different subsets of the stability-ranked list, using STEM software [31]. The functional categories that were significantly enriched among the top 1000 IL-3 responsive transcripts are listed in Supplemental Table S3. IL-3 disproportionately decreased the turnover of transcripts annotated to the GO categories cell cycle (p<2×10^−10^), RNA processing (10^−6^), and other related categories. As further support for coordinated function of the IL-3-stabilized genes, Ingenuity pathway analyses linked five out of the top fifteen stabilized transcripts (and 25 out of the top 250 stabilized transcripts) into a proliferation-related interaction network (data not shown). This network was scored at a highly significant level of 47, using algorithms that assign a score of 2 to a 1% likelihood of random association [32].

### Candidate motifs involved in IL-3-mediated stabilization

**Figure 3:**
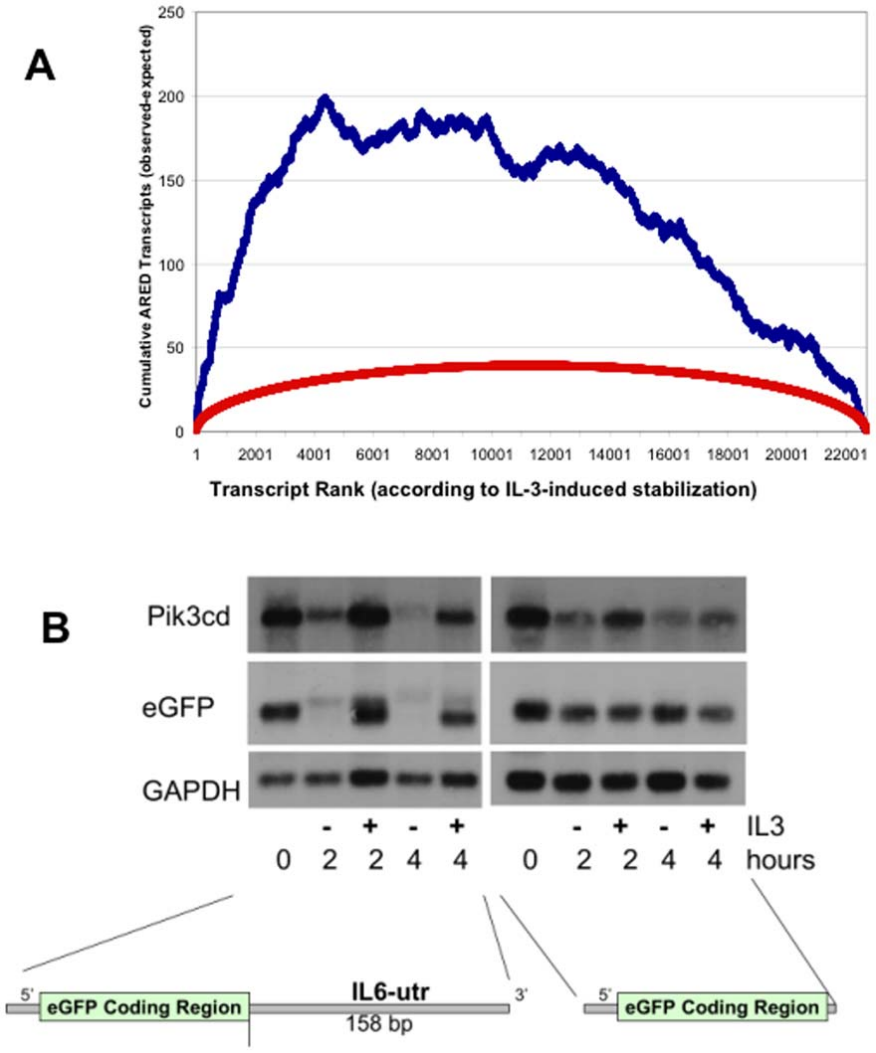
Contribution of AU-rich elements to IL-3 control of transcript stability. *A. Enrichment for ARED sequences in IL-3-stabilized transcripts*. The excess accumulation of AU-rich transcripts as a function of position in the ranked list is shown (blue). An upward slope indicates that AU-rich sequences are accumulating at a faster rate than predicted by random ranking; a downward slope indicates that ARE's are accumulating at a slower rate than random transcript ranking would predict. The 95th percentile confidence interval curve for random rankings is also plotted for comparison (red). *B. IL-3 response element mapping in the IL6 promoter*. A 158-bp IL6-3′-UTR fragment destabilizes a heterologous transcript in the absence, but not in the presence, of IL-3. Northern blotting of RNA from 32Dcl3 cells stably transfected with EGFP or an EGFP-IL6UTR transgene. GAPDH indicates loading and pik3cd demonstrates endogenous transcript regulation in the presence and absence of IL-3. Representative blots of two independent experiments are shown.

A bioinformatics approach was used to uncover common motifs among the transcripts ranked by differences in their turnover rate with and without physiologic IL-3. As a first indication of whether IL-3 was acting through discrete motifs, we determined whether AU-Rich Element (ARE)-containing transcripts were overrepresented among those stabilized by IL-3. The ranked transcript dataset derived from our kinetic microarrays was plotted against the ARED database version 3.0 [33] that is comprised of genes reported to have functional AU-rich sequence elements in their 3′-UTR. The set of AU-rich genes was defined based on the presence in ARED database 3.0 [33]. As the ARED database contains human genes, mouse genes were mapped to a human gene if a homolog relationship was present in the homologene.data file provided by the National Center for Biotechnology Information (NCBI). The presence or absence of ARE's logged into ARED is noted individually for each probe set in our database (Supplemental Table S4). The difference in the cumulative number of observed matches with the ARED database based on ranking in our list and the expected number if we had randomly ordered our list was calculated. The lower curve in Figure 3A shows calculated AU-rich accumulation rates if the AU-sequences had been randomly distributed. The 95^th^ percentile curve for AU-accumulation for randomly-ordered probesets is shown. We observed increasing concentration of AU-sequences in probe sets corresponding to the first 4000 transcripts, with the steepest increase among the transcripts that were most stabilized by IL-3, particularly in the first 780 transcripts. This is evident by the slope of *cumulative* observed-minus-expected ARED transcripts as shown in Figure 3A. The steeper the positive slope, the higher the concentration of AU-containing transcripts among the corresponding probe sets. Based on the hypergeometric distribution the p-value enrichment for ARED transcripts among the first 250 transcripts is <10^−9^ and among the first 4000 transcripts it is <10^−22^. Similar results were obtained when unique transcripts were ranked (averaging values of corresponding probe sets) and used as when the analysis included all probe sets (data not shown). It should be noted that while AU-rich transcripts tend to be short-lived, there was no *a priori* reason why the rapid decay of these mRNAs would be inhibited by IL-3. The observation of ARED clustering among IL-3-stabilized transcripts suggested that IL-3 signaled through pathway(s) that altered mRNA regulation at specific sequences associated with AU-binding proteins.

In order to validate the importance of ARE-containing sequences in transcripts stabilized by IL-3, we investigated whether IL-3 responsiveness mapped to a domain containing AU-sequences within the 3′-UTR of interleukin-6. The IL-6 gene contains the shortest 3′-UTR (420 bp) of those murine transcripts whose stabiliization by IL-3 was validated in Figure 2. We therefore constructed a fusion construct that linked EGFP cDNA to an ARE-containing 158-bp murine IL-6 3′-UTR domain that contains 4 AU-rich elements. This construct or an EGFP control plasmid were stably transfected into 32Dcl3 cells. EGFP turnover was subsequently monitored using Actinomycin D chase assays. The IL6-UTR domain conveyed IL-3-dependent turnover to the EGFP transcript as shown in Figure 3B.

### IL-3 signaling pathways involved in transcript stabilization

**Figure 4:**
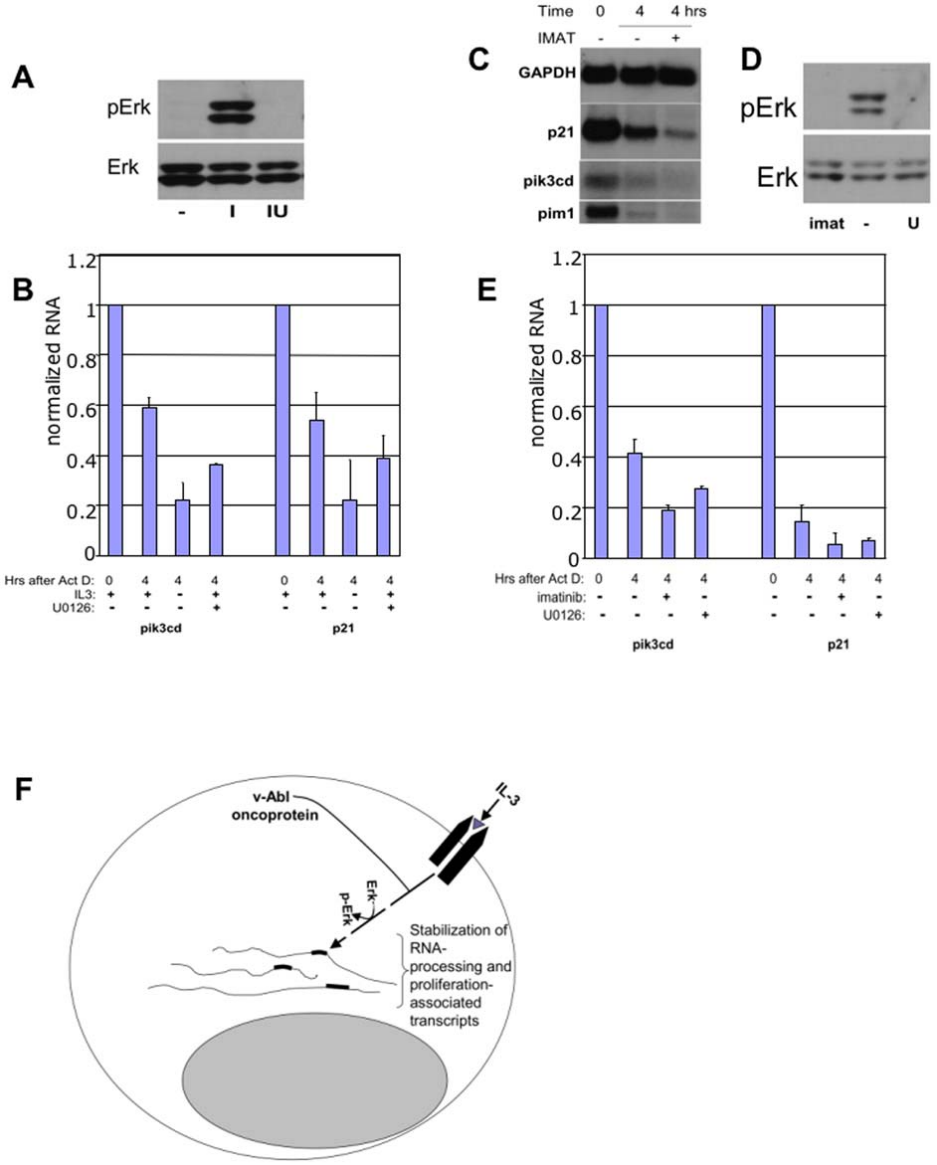
IL-3-mediated RNA stabilization involves MEK/ERK signaling. *A. IL-3 activates pErk in 32Dcl3 myeloblasts*. Upregulation of phospho-Erk at 15 minutes after addition of IL-3 to 32Dcl3 myeloblasts washed free of IL-3 is evident (I). Pre-incubation for 20 minutes with the Mek1 inhibitor U0126 (at 10 uM) blocks this effect (IU), as does washout of IL3 (−). *B. Downregulation of IL-3 induced transcript stabilization by Mek/ERK blockade*. Cells were incubated with 10 uM of 10 uM of erk inhibitor U0126 concurrent with Actinomycin D. 1 ng/ml IL-3 (+) or vehicle was added 20 minutes later (time 0) and RNA was harvested 2 or 4 hours after IL-3 addition. p21 and pik3cd expression on Northern blots normalized to GAPDH is shown, with time 0 value set to 1.0 (n = 3). *C. Imatinib inhibits mRNA stability in v-Abl 32Dcl3 cells*. Imatinib (IMAT) or DMSO vehicle (−) were added following a 20 minute preincubation with actinomycin D, at time 0 as indicated. Cells were harvested for RNA at 0 and 4 hours and Northern Blots were probed with pik3cd, p21, pim1 and GAPDH. *D. Erk phosphorylation*. Phosphorylation of Erk in v-Abl transformants of 32Dcl3 is prevented by 15 minute incubation with imatinib (I, 5 uM) or with U0126 (U, 10 uM). *E. Effects of imatinib and U0126 on transcript turnover*. Data represents three separate experiments. Quantitation of RNA expression on Northern blots normalized to GAPDH, with time 0 value set to 1.0 is shown. *F. Model of posttranscriptional pathway*. Dashed line indicates possible involvement of additional signals.

IL-3 signals through multiple pathways to support hematopoietic cell growth and survival. Among the pathways and kinases activated by IL-3 in cytokine-dependent cell lines such as 32Dcl3 are PI3K, Jak2/Stat5, PKC, src, ras/raf/mek/erk,MAPK/p38 or Jnk pathways (for review [34]). Activation of each of these pathways has been associated with mRNA turnover control (reviewed in [35]). In order to garner insight into pathways through which IL-3 could coordinately stabilize myeloblast transcripts, we separately inhibited PI3K, JAK/Stat, PKC, Mek/Erk, Jnk, Src, Syk and AMPK signaling pathways using optimized inhibitor concentrations and determined whether any of these pathways modulated IL-3-induced transcript stabilization. Inhibitors were added 15–20 minutes before IL-3 addition in Actinomycin D chase assays. Of these inhibitors, only the Mek1 inhibitor U0126 decreased the ability of IL-3 to stabilize transcripts. This inhibitor was used at a concentration of 10 uM that sufficed to block Erk phosphorylation in these cells (Figure 4A). Because U0126 and subsequently IL-3 were added after transcriptional blockade, these results indicated a role for Erk signaling in the posttranscriptional stabilization of transcripts by IL-3 (rather than an effect on transcription). The Erk pathway was of particular interest because it has previously been linked to posttranscriptional stabilization of p21, IL-8 , MIP 1alpha, IL-1beta, TNFalpha, cox-2 and GM-CSF mRNAs [36]–[42] (see also commentary by Sugiura [43]). However, inhibition of the MEK/Erk pathway partially blocked but did not completely abnegate transcript stabilization by IL-3 (Figure 4B).

### Abl kinase recruitment of IL-3 posttranscriptional stabilization pathway

Conceivably, oncoproteins could hijack posttranscriptional pathways normally used by growth factors to sustain proliferation and prevent differentiation of immature cells. 32Dcl3 myeloblasts transduced with the v-Abl oncoprotein were used to test whether this constitutively-active kinase affected mRNA turnover and shared mRNA targets with IL-3. We have shown that v-Abl renders 32Dcl3 cells IL-3 independent for growth and blocks differentiation [2]. Figure 4C shows that active abl kinase (compared with imatinib-inhibited v-Abl) increased the half-life of transcripts included among IL-3-stabilized transcripts profiled in Figure 2. As in the case of IL-3, active v-Abl promoted Erk phosphorylation in 32Dcl3 myeloblasts (Figure 4D). Inhibition of Erk phosphorylation by the Mek1 inhibitor U0126 partially blocked v-Abl stabilization of transcripts in comparison with imatinib (Figure 4E).

## Discussion

Changes in the stability of messenger RNAs represent an efficient way rapidly to alter gene expression and cellular phenotypes, particularly if multiple mRNAs that share a functional pathway are stabilized or destabilized in synchrony. Coincident increases (or decreases) in mRNA transcription and in mRNA stability can magnify signals, whereas discordant transcription and mRNA stability enforces precise temporal or developmental regulation of transcript levels. Extensive research has delineated transcriptional control pathways during myelopoiesis that are disrupted in leukemia and more recent investigations have highlighted differentiation-specific or leukemia-related posttranscriptional modulation of translation by microRNAs. The impact of mRNA stability control on normal and malignant myelopoiesis has been less well characterized despite evidence that changes in turnover rates of certain mRNAs can dramatically affect cellular behavior (for review, [35]).

This paper delineates the range of posttranscriptional mRNA regulation by IL-3 in murine myeloblasts as evidence that IL-3 controls myeloblast function through its effects on mRNA turnover rather than solely through transcriptional activation. The population of IL-3-stabilized transcripts was captured through analysis of the decay rate of transcripts in IL-3 -replete or -deficient medium after transcriptional blockade with Actinomycin D. While mRNA half-life changes have previously been studied in LPS-exposed monocytes, activated T cells, flavopiridol-exposed lymphocytes and HepG2 cells [18], [44]–[46], a systematic study of growth factor-directed transcript turnover in myeloblasts has not hitherto been conducted. We have previously reported a robust bioinformatics approach to short kinetic gene expression data sets [47] and in this study we have extended that work to optimize the comparison of microarray sets charting transcript decay under two discrete conditions. The algorithm that we used to compare global mRNA decay rates under these conditions will be useful for other kinetic microarray analysis of mRNA stability.

This analysis ranked transcripts from most- to least-stabilized by IL-3. Northern blotting of cells incubated with or without IL-3 confirmed IL-3 mediated stabilization of select transcripts ranked from position 3 to position 273 out of 22,690 probe sets on an Affymetrix array (Table 1, Figure 2). The number of transcripts whose decay rate is slowed by IL-3 is therefore substantial, supporting the relevance of this posttranscriptional pathway to IL-3-supported survival, growth and differentiation blockade of 32Dcl3 myeloblasts. Many of the proteins encoded by IL-3 stabilized transcripts have been shown to support leukemic cell growth and survival and/or to inhibit differentiation in 32Dcl3 myeloblasts (for examples, Table 1). These results supported a significant role for IL-3-mediated RNA stabilization in sustaining myeloblast growth and suggested that elucidation of IL-3-stabilized transcripts could reveal genes and pathways that support blast cell proliferation in myeloid leukemias. In this context, it was notable that two of the five most stabilized genes, pik3cd and IL6 are already directly targeted by therapeutics in clinical trials of hematologic malignancies (NCT00710528, NCT00402181).

It is notable that transcripts involved in RNA processing were significantly enriched as IL-3 stabilization targets upon gene ontology analysis. Enhanced expression of this class of genes could serve to bolster IL-3 effects on decay rates, or alternatively, to limit IL-3 posttranscriptional effects. Two transcripts that were markedly stabilized by IL-3, BRF1 (zfp36l1) and BRF2 (zfp36l2), destabilize mRNA transcripts at AREs [48], [49]. These gene products have been linked to leukemia and may facilitate degradation of apoptotic or antiproliferative transcripts in myeloblasts. Alternatively, BRF1 and 2 transcript stabilization could herald a negative feedback loop that limits the durability of IL-3-sustained messages. This possibility is supported by a recent global assessment of epidermal growth factor signaling that identified early upregulation of BRF1 and 2 as part of a negative feedback loop that was characterized by BRF destabilization of mitotic transcripts [50].

Our data indicated that some of the same transcripts stabilized by IL3 are stabilized by the v-abl oncoprotein in an imatinib-dependent manner. This raises the prospect that many of the growth-promoting mRNAs that are stabilized by IL3 are also stabilized by v-Abl, potentially through the same terminal mediator(s). Such a mechanism could contribute to the ability of v-Abl to substitute for IL-3 in supporting the growth of this myeloblast cell line. Because multiple oncogenes can render these myeloblasts factor-independent, it is conceivable that the appropriation of growth factor pathways regulating RNA decay is a general feature of leukemogenesis. IL-3 and v-Abl share overlapping signaling pathways, including activation of the Mek/Erk pathway. This pathway has been shown to stabilize several transcripts (as discussed in Sugiura [43]). In our experiments, the Mek inhibitor U0126 partially inhibited IL-3 and v-Abl-mediated transcript stabilization. This suggests that Erk cooperates with other pathways downstream of IL-3 and v-Abl to stabilize target mRNAs, as has been reported for Erk-mediated TNF-alpha transcript stabilization [40].

Studies are planned to dissect in detail the downstream mediators of IL-3 and v-Abl posttranscriptional control of mRNAs in order to facilitate the development of “post-transcriptional” therapeutics for leukemia. Such therapies could be directed at normalizing the turnover of aberrantly stabilized mRNAs by targeting pathways or protein/RNA interactions responsible for coordinated transcript stabilization in malignancy.

## Materials and Methods

### Cells and Culture

32Dcl3 cells (gift from Alan Friedman, Johns Hopkins University) were cultured in RPMI 1640 media supplemented with 10% fetal bovine serum as previously described [27] using murine IL-3 (Peprotech, Rocky Hill, NJ) at 1.5 ng/ml during cell passaging. To generate stable clones lacking UTR sequences, 32Dcl3 cells were transduced with retroviral supernatants prepared to express human p21 from an engineered pMig construct (MIGhp21wt) as described below. Transduced cells were sorted on the basis of GFP expression. 32Dcl3 cells expressing the entire human p21 gene were transfected with pC-WAF1-S (gift from Wafik El-Deiry, U of Pennsylvania) and selected in hygromycin. To generate stable clones bearing eGFP with or without IL6- UTR sequences, cells were electroporated using Amaxa electroporation per manufacturer's guidelines (Amaxa, Inc., Gaithersburg, MD) for 32Dcl3 cells, using 10^−6^ cells and 2.0 ug DNA, followed by neomycin selection. V-Abl cells were generated as previously described [2].

### Plasmids

Retroviral constructs were derived from the MIG plasmid (gift from Luk Van Parijs, Massachusetts Institute of Technology) which we modified into a Gateway® destination vector (Invitrogen, Carlsbad, CA). Human p21 wild-type cDNA (accession# NM_078467) coding region carrying a myc-his tag was cloned into this using Gateway® technology. To generate pEGFP-IL6-UTR, the plasmid pEGFP-N1 (Clontech, Inc., Mountainview, CA) was digested at the Not1 site, dephosphorylated, and ligated with a PCR-generated fragment encompassing bp 740–898 of murine IL6 (Accession NM_031168) generated with Eag1 and Not1 bounded primers that was digested with Eag1 prior to ligation.

### Retroviral Transduction

Retroviruses were generated and transduced into 32Dcl3 cells as previously described [51].

### RNA Half-life determination

32Dcl3 cells were washed free of IL-3 and resuspended in IL-3-free medium. Actinomycin-D (Sigma-Aldrich) was added at 10 µg/mL, a concentration that we have previously shown to suppress transcription in 32Dcl3 cells [2]. Twenty minutes after Actinomycin D addition, the culture was divided into aliquots for growth factor addition. IL-3 was added at a final concentration of either 1 ng/mL, 10 pg/mL or absent and RNA was prepared at time points as noted. For half-life assessments made in G-CSF, cells were washed free of IL-3 and incubated in G-CSF for 18 hours. Actinomycin D was added at time −15 minutes and cell cultures were divided into two equal parts. Cells in G-CSF received either 1 ng/mL IL-3 or no cytokine 15 minutes after Actinomycin D addition. RNA was prepared at denoted time points after cytokine addition. Northern blotting for all experiments was performed as previously described [27]. Signal density analysis of scanned images was performed using Image J 1.31v software (http://rsb.info.nih.gov/ij). Multiple exposures were obtained to optimize linearity of signal. Half-life determination was calculated from logarithmic trendlines fitted to the plotted data of signal magnitude (normalized to time 0) versus time.

### Riboprobes

Templates for riboprobes were generated using RT-PCR of mRNA purified from 32Dcl3 cells or by amplification from plasmid templates using T7-linked antisense oligomers. A full listing of PCR sequences used to amplify probes is available on request. ^32^[P]-UTP labeled riboprobes were generated *in vitro* using a T7-Maxi-Script kit (Ambion, Houston, TX) per manufacturer's instructions.

### Statistics

Protein and RNA half-life determinations were compared for all conditions tested with significance assessed by a 2-tailed, type 2 student's t-test using 95% confidence intervals. All calculations were made using the Excel software package (Microsoft, Redmond, WA).

### Gene Ontology Analysis

We analyzed the top 1000 probe sets for enrichment of the corresponding genes belonging to common Gene Ontology (GO) categories. The base set of genes was all genes on the microarray. The GO analysis was done using the STEM software [31] the p-values based on the hypergeometric distribution. GO enrichment p-values were corrected for multiple hypothesis testing using randomization analysis.

### Microarray Normalization

Most microarray normalization methods assume that the total mRNA quantity will remain constant across the different arrays and time points. This assumption is not applicable to the kinetic microarray experiments conducted to measure mRNA decay, because later time points followed transcriptional blockade. We thus evaluated several normalization programs (dChip, MAS 5, and RMA) to determine which would be most consistent with our expectation that the mRNA levels at time point 0 would contribute a greater signal than time points after transcriptional inhibition. Using thiscriteria we selected the dChip normalization (see Supplemental Methods S1 for details). All ten microarray hybridizations were normalized together using the dChip software with the default settings.

### Scoring Probe Sets

The probe sets were ranked for IL-3 induced stability based on the signed area between a curve for the high IL-3 experiment and the low IL-3 experiment. For each probe set, the signed area values were averaged over the two replicates and divided by a variability term. The variability term was a function of the average intensity of the probe set for the 0 hour time point. This function was computed based on the variability of the area between the replicates for probe sets with similar average 0 hour probe set intensity. See Supplemental Methods S1 for full details.

### Data Accession

Microarray data is available at http://www.ncbi.nlm.nih.gov/geo/query/acc.cgi?acc=GSE12067.

all microarray data reported in the manuscript is described in accordance with MIAME guidelines.

## Supporting Information

Supplemental Methods S1 Detailed methods related to microarray normalization and probeset ranking.
(0.62 MB DOC)

Supplemental Table S1 Affymetrix 430A probe sets ranked from most stabilized by IL-3 to least stabilized according to normalized area score. Gene symbols and identifiers are included.
(3.02 MB XLS)

Supplemental Table S2 Unique transcripts according to Entrez ID ranked from most stabilized by IL-3 to least stabilized according to normalized area score. Score shown is the average score of all probe sets that were linked to the given transcript. Gene symbols and Entrez ID identifiers are included.
(1.12 MB XLS)

Supplemental Table S3 Gene Ontology Analysis conducted on the 1000 transcripts most-stabilized by IL-3. Analysis conducted as described in text.
(0.05 MB PDF)

Supplemental Table S4 Excess occurrence of ARE motifs among transcripts most stabilized by IL-3. See text for details. Sheet 1 lists all probe sets from most stabilized (position 2) to least stabilized (position 22691). A value (“1”) is ascribed to each probe set with a human match to the ARED database (Reference 26) and a “0” value to those lacking a match. Cumulative ARED-containing transcripts in the rank list compared to random ordering (observed minus expected) is derived from this chart as discussed in the text.
(4.02 MB XLS)

Figure S1 Schematic of data collection for global transcript decay analysis. Following actinomycin D addition, cells were cultured in IL-3 replete or deficient medium starting at time 0. In followup Northern Blot experiments, pathway inhibitors were added 15–20 minutes before IL-3 or vehicle control was added.
(0.07 MB TIF)

Figure S2 Top: Stability plots of myeloblast transcript probe sets determined using kinetic microarrays. In these plots, the garea value (y-axis) is plotted against the intensity of the probe signal at time 0 (x-axis. Increased stability correlates with positions farthest from the origin. The highest ranked 250, 1000, or 2000 probe sets based on gscore as well as all probe sets with positive garea values (5290 total) are shown and highlighted by color. The plots of the top 250 Blue), 1000 (red), and 2000 (green) and all positive probe sets (grey) in terms of the difference between their decay curve in IL-3 -replete and -deficient medium are overlaid. The top 15 ranking probe sets are labeled with their corresponding gene names. The identities and ranking of all 22,690 probe sets can be found in Supplemental Table 1. The top 1000 probesets account for 53% of the total cumulative garea attributable to IL3-stabilization. Bottom: The difference between transcript half-lifes in IL3 and in IL3-deficient conditions (normalized area score) is plotted for 22690 probe sets arranged from most stabilized by IL-3 (positive values) to least stabilized by IL-3 (negative values; less stable in IL-3). The broken axis facilitates notation of the last probeset (rank 22690) with a value of −148. The region corresponding to the indicated probesets on the top graph is circled.
(0.17 MB TIF)
