# Supplementary material for: IL-3 and Oncogenic Abl Regulate the Myeloblast Transcriptome by Altering mRNA Stability
Source: PLoS One. 2009 Oct 15;4(10):e7469. doi: 10.1371/journal.pone.0007469 (PMC2758590; doi:10.1371/journal.pone.0007469)
Supplement: Supplemental Methods S1 — Detailed methods related to microarray normalization and probeset ranking. (0.62 MB DOC) [file pone.0007469.s001.doc]

**Supplemental methods**

SM1. *Normalization of microarray*

The dChip software ([www.dchip.org](http://www.dchip.org/))^1^ with the default settings was used to normalize the microarray data. All ten hybridizations which comprised duplicate analyses of five timepoints from two independent experiments were normalized together as one batch. Two other normalization methods, RMA^2^ and MAS 5 ^3^, were also considered. However as shown in Figure S1, the dChip method was selected as it was most consistent with the experimental expectation that the time point 0h experiment should represent a greater percentage of the total the sum of the signals observed on the probe across all experiments in the replicate. The reason for this expectation is that transcription was shut down during the other experiments. A concern with normalization methods for this experimental design is that they would mistake the biologically expected global expression difference between experiments to be a result of systematic noise and then severely reduce it. This appears to be less of a concern with the dChip method as compared to the other methods.


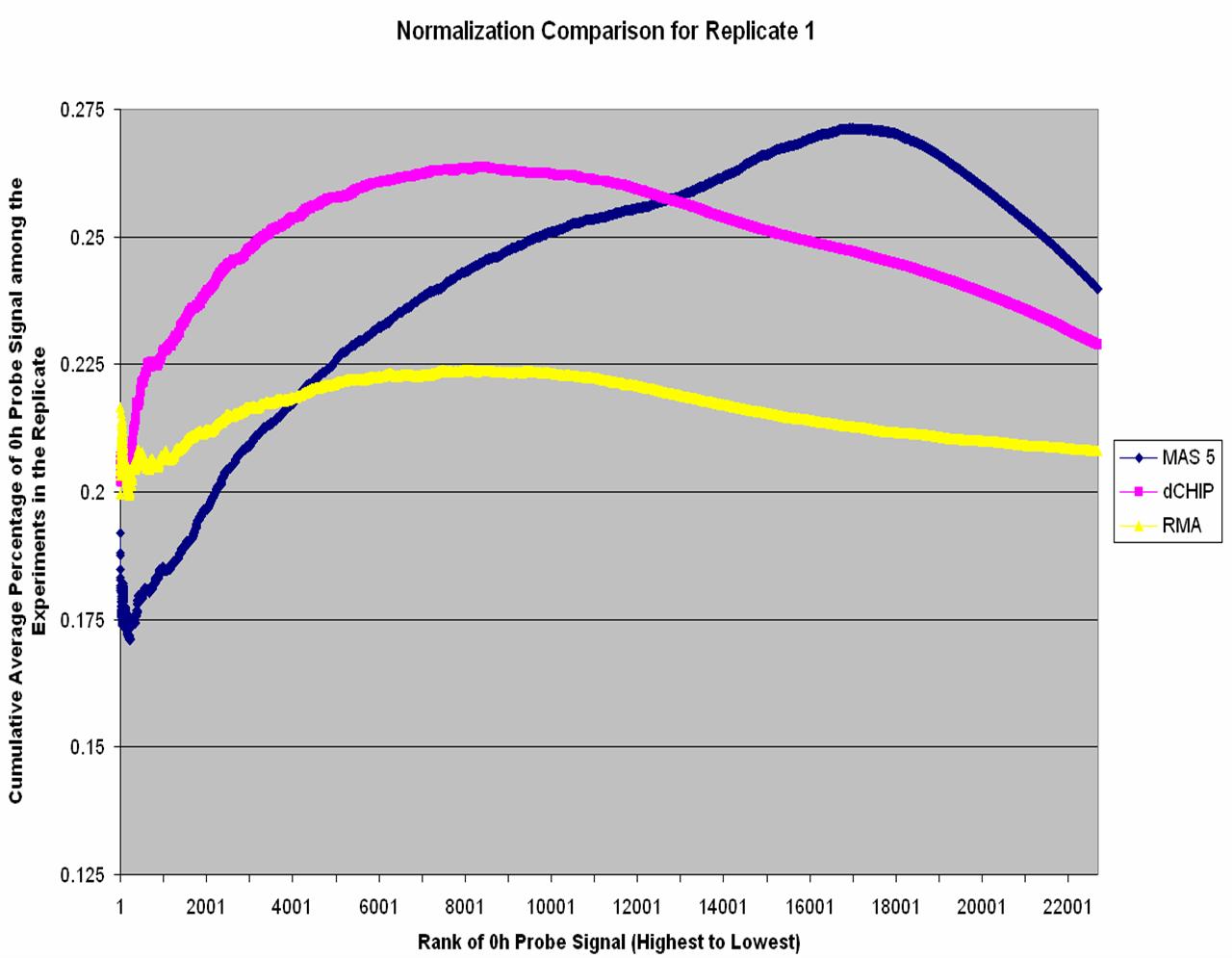

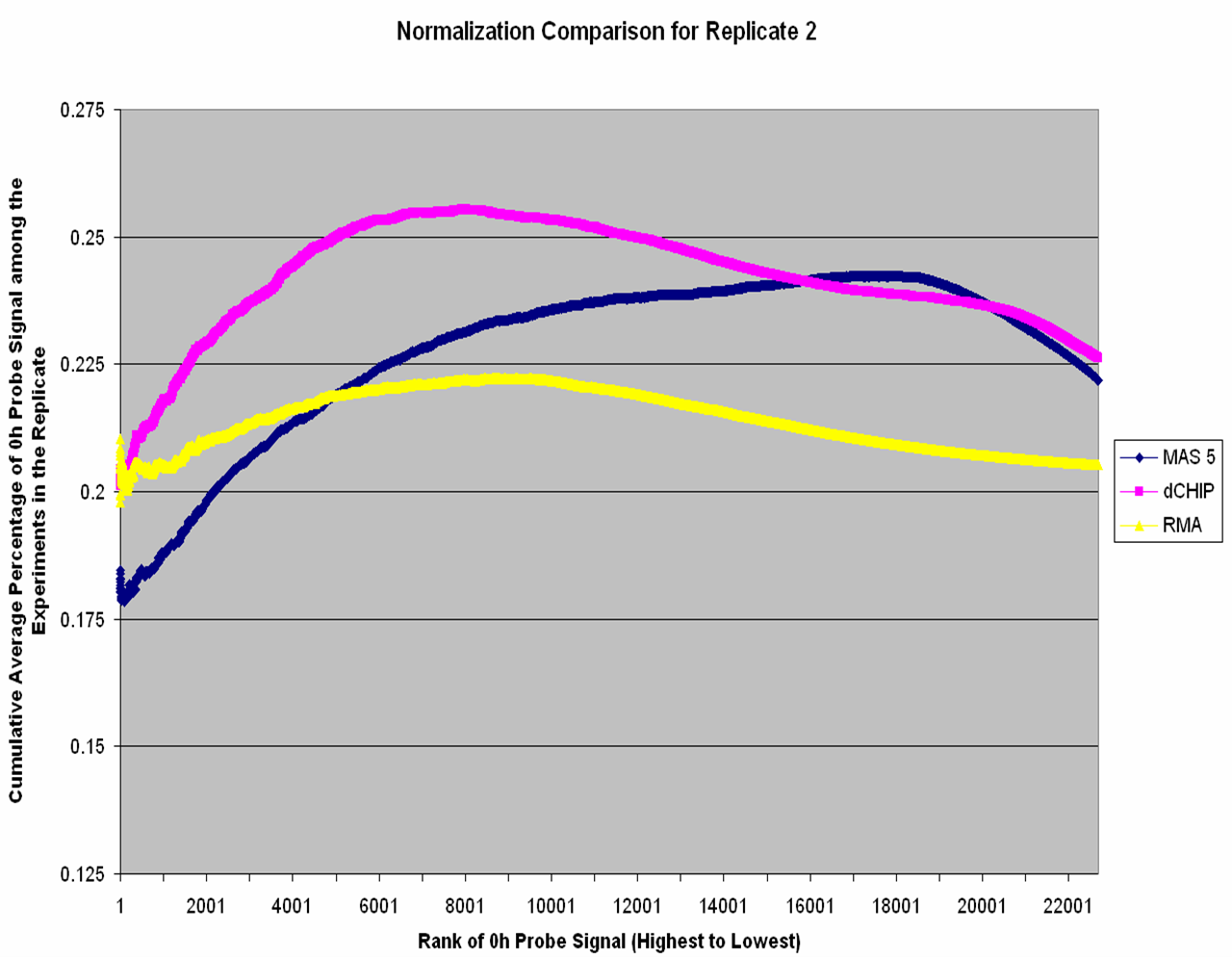


Figure S1: A comparison of the dChip, MAS5, and RMA methods separately on each replicate. The graphs plot for each normalization method the cumulative average percentage of the total signal for a probe set that was given to the 0h experiment among all experiments in the replicate. If the signal was equally distributed across all experiments then a value of 0.2 would be obtained. The dChip normalization was most consistent with the experimental expectation that on average the 0h time point should represent a greater percentage of total expression of each probe.

SM2. Probe Set Ranking Method

The following method was used to rank probe sets for having the greatest evidence for increased mRNA stability in response to high IL-3 treatment as compared to low IL-3 treatment of its corresponding gene. Let *gtreatment_time_R* denote the dChip normalized expression value of probe set *g* for the hybridization with IL-3 treatment type of *treatment*$\in${*none*, *low*, *high*}, sampled at time point *time*$\in${*0h,2h,4h*}, from replicate *R* where *R*$\in${1,2}. We first defined for each probe set *g* and replicate *R*, a quantity *garea_R*, as

$$g_{area\_R}=2\times\left(\min\left(\frac{g_{high\_2h\_R}}{g_{none\_0h\_R}},1\right)-\frac{g_{low\_2h\_R}}{g_{none\_0h\_R}}\right)+\left(\min\left(\frac{g_{high\_4h\_R}}{g_{none\_0h\_R}},1\right)-\frac{g_{low\_4h\_R}}{g_{none\_0h\_R}}\right)$$

The above expression can be derived by first taking the ratio of each value with the time point 0 value from its replicate. Next each ratio value from a high IL-3 experiment that was above one, was set to one. This step avoids giving extra preference to probe sets whose expression value was higher than the time point 0h experiment as compared to having the same value as it (since transcription was blocked, any value higher in the 2h and 4h time point is the result of measurement noise) . The resulting transformed values are then used to create two curves, a high curve and a low curve (see Supplement Figure 1). The high (low) curve is created by using two line segment to connect the 0h, 2h high (low) points, 4h high (low) transformed values. The formula above then corresponds to the signed area (the integral) between the high and low curves during the period between 0h and 4h.


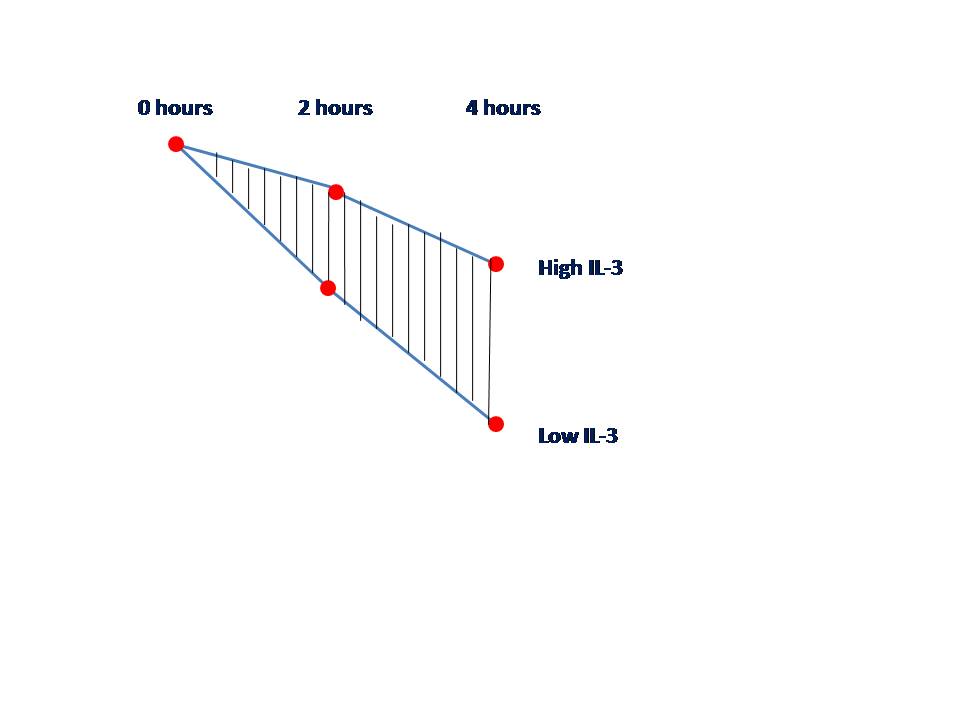


Supplement Figure 1: Computation of the g_area_ value. The high and low IL-3 curves were plotted by taking the ratio with respect to time point 0. The g_area_ value is the area between the two curves (black lines between the blue curves).

We note that in the formula small additive amounts of noise for probe sets with relatively low signal could have relatively large effects on *garea*_*R*. Thus many of the highest ranking probe sets based on *garea_R* could be explained as a result of noise on low signal intensity probe sets. Instead of ranking probe sets based on *garea_R* we rank probe sets based on how many times greater *garea_R* is than an average measure variability we associate with probe sets of similar 0h intensity.

To define our variability measure we first re-define the area formula from above to eliminate the *min* operation, since that causes some probe sets to artificially have no variability associated with them. The re-defined formula for a probe set *g* whose expression is denoted as *g_area_*'_*R* is

$$g_{area'\_R}=2\times\left(\frac{g_{high\_2h\_R}}{g_{none\_0h\_R}}-\frac{g_{low\_2h\_R}}{g_{none\_0h\_R}}\right)+\left(\frac{g_{high\_4h\_R}}{g_{none\_0h\_R}}-\frac{g_{low\_4h\_R}}{g_{none\_0h\_R}}\right)$$

Our variability measure associated with a probe set will be a function of the average of the 0h probe set intensities in the two replicates. To do this first we define the quantity

$$v_{i}=|g_{area'\_1}^{i}-g_{area'\_2}^{i}|$$

where$g^{i}$ is the probe set for which the quantity$\frac{g_{none\_0h\_1}^{i}+g_{none\_0h\_2}^{i}}{2}$is the *i*^th^ largest among all probe sets we consider for this variability estimation (in case of ties probe sets with larger *v_i_* values go first). We considered all probe sets except for six outliers.

We next define a function *W* that computes for a ranking *j* based on average 0h signal intensity the average of all *v_i_* values within 500 places of it in either direction (fewer values are used in the average at the extreme ends), that is

$$W\left(j\right)=\frac{\sum_{i=\max(1,j-500)}^{\min(j+500,N)} v_{i}}{1001-\max(j+500-N,0)-\max(501-j,0)}$$

Where *N* is the total number of probe sets being considered. While this function generally decreases (see Figure 2), to ensure that this function decreases monotonically we define a function *V* as

$$V\left(j\right)=\frac{\min_{k=1:j}(W(k))+\max_{k=j:N}(W(k))}{2}$$

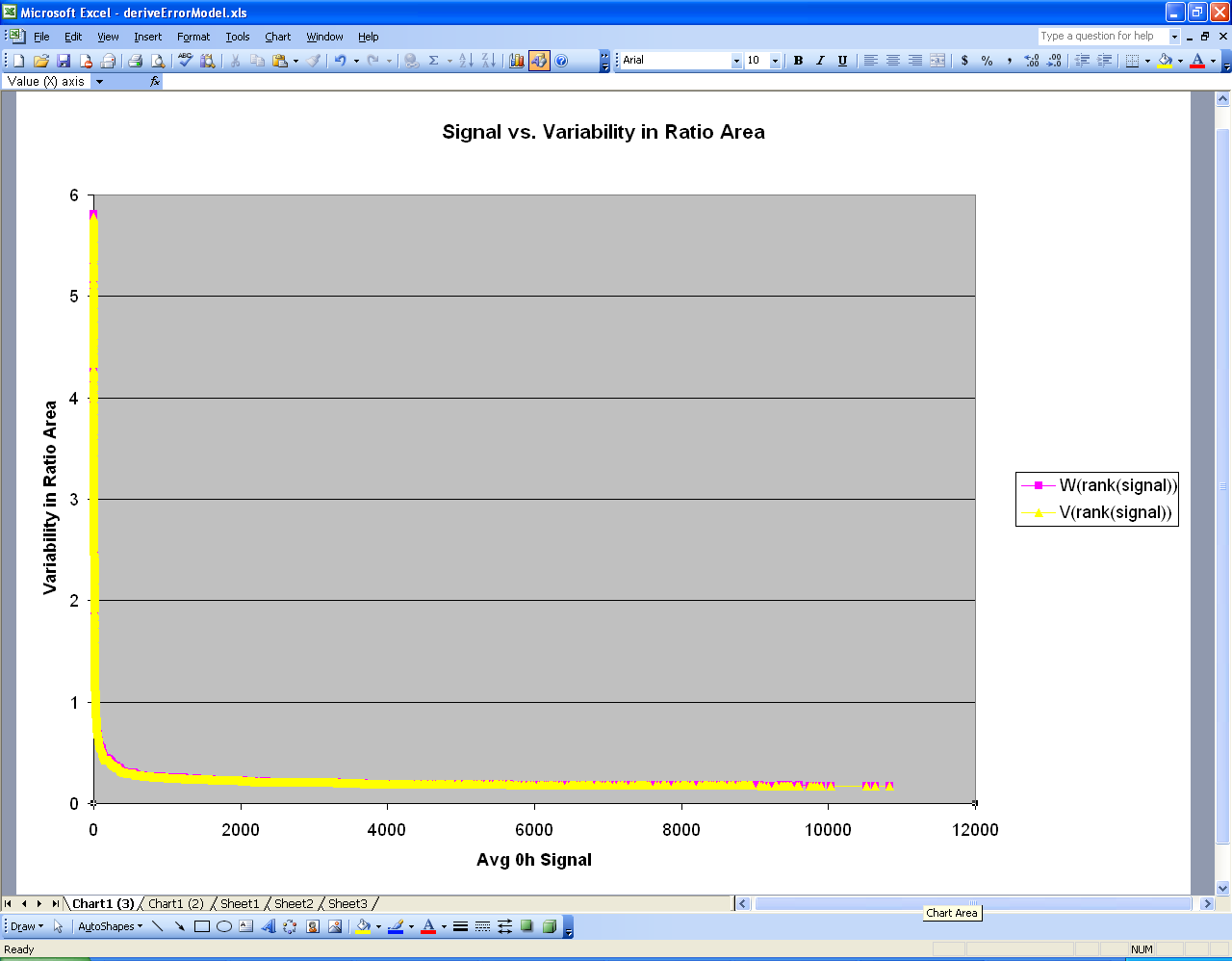

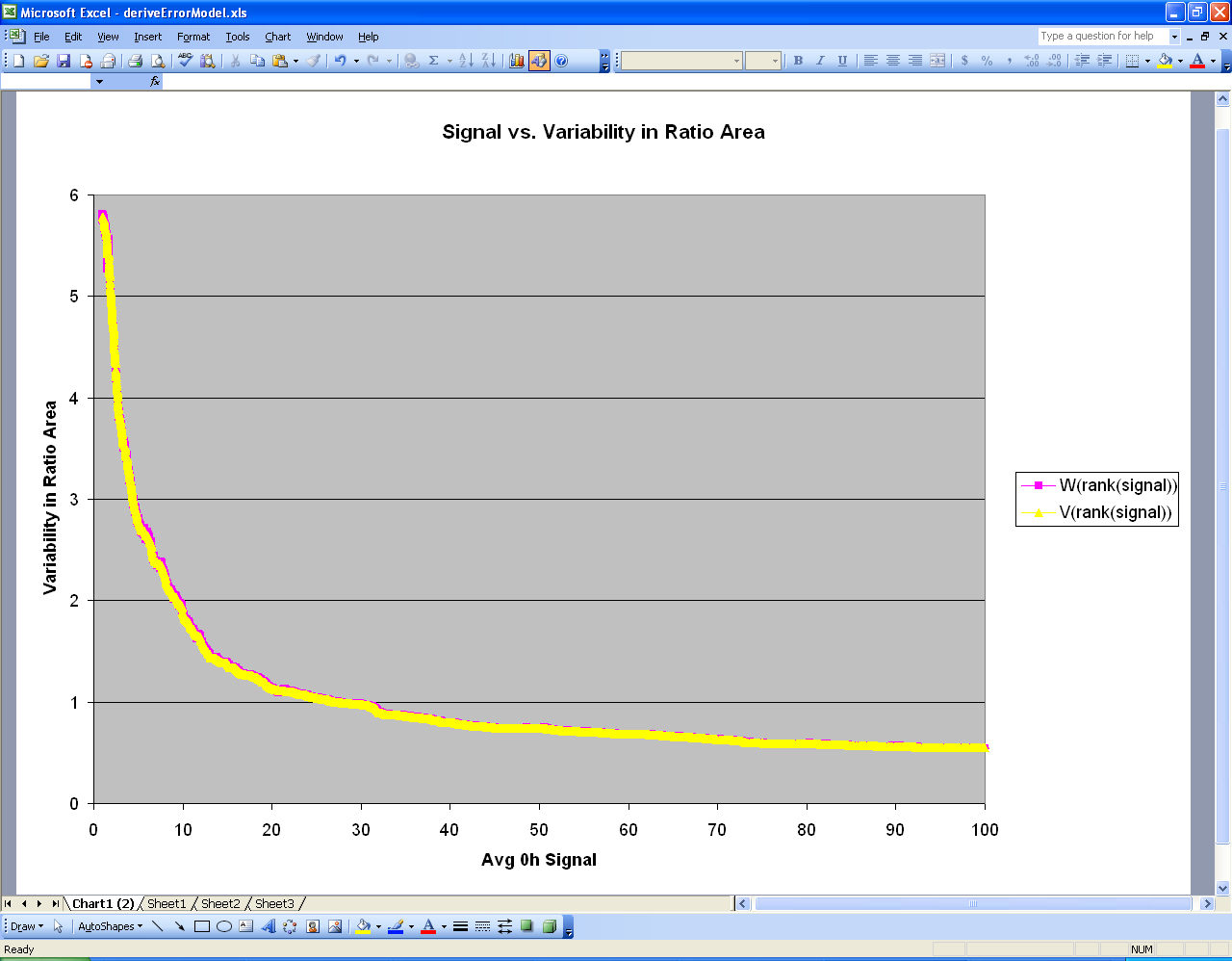


Figure S2: The x-axis is the average signal intensity of the 0h time point. The y-axis is our variability measure. The pink line represents our function *W*, the yellow line represents the montonically decreasing adjustment, *V*. The image on left shows the entire range of avg 0h signal intensity, while the image on right zooms on signal intensity less than 100.

Finally we are able to define the score of a probe set as

$$g_{score}=\left(\frac{g_{area}}{V\left(rank\left(\frac{g_{none\_0h\_1}+g_{none\_0h\_2}}{2}\right)\right)}\right)$$

where

$$\mathop{g_{area}}\limits=\frac{g_{area\_1}+g_{area\_2}}{2}$$

and *rank*(*x*) is the maximum index *j* such that this expression is minimized

$$\left|x-\frac{(g_{none\_0h\_1}^{j}+g_{none\_0h\_2}^{j})}{2}\right|$$

Supplemental References

1. Li C, Wong WH. Model-based analysis of oligonucleotide arrays: expression index computation and outlier detection. Proc Natl Acad Sci U S A. 2001;98:31-36.

2. Bolstad BM, Irizarry RA, Astrand M, Speed TP. A comparison of normalization methods for high density oligonucleotide array data based on variance and bias. Bioinformatics. 2003;19:185-193.

3. Hubbell E, Liu WM, Mei R. Robust estimators for expression analysis. Bioinformatics. 2002;18:1585-1592.
