# Supplementary material for: IL-3 and Oncogenic Abl Regulate the Myeloblast Transcriptome by Altering mRNA Stability
Source: PLoS One. 2009 Oct 15;4(10):e7469. doi: 10.1371/journal.pone.0007469 (PMC2758590; doi:10.1371/journal.pone.0007469)
Supplement: Supplemental Table S3 — Gene Ontology Analysis conducted on the 1000 transcripts most-stabilized by IL-3. Analysis conducted as described in text. (0.05 MB PDF) [file pone.0007469.s004.pdf]

Category ID	Category Name	#Genes Category	#Genes Assigned	#Genes Expected	#Genes Enriched	p-value	Corrected p-value
GO:0044424	intracellular part	13399	716	590.5	125.5	2.40E-17	<0.001
GO:0005622	intracellular	13868	728	611.2	116.8	1.50E-15	<0.001
GO:0005634	nucleus	6445	397	284	113	2.60E-15	<0.001
GO:0043231	intracellular membrane-bounded organelle	10278	556	453	103	1.50E-11	<0.001
GO:0043227	membrane-bounded organelle	10291	556	453.5	102.5	2.00E-11	<0.001
GO:0007049	cell cycle	1120	97	49.4	47.6	1.30E-10	<0.001
GO:0000278	mitotic cell cycle	484	53	21.3	31.7	1.10E-09	<0.001
GO:0043229	intracellular organelle	11513	599	507.4	91.6	1.70E-09	<0.001
GO:0016071	mRNA metabolic process	507	51	22.3	28.7	3.90E-08	<0.001
GO:0000087	M phase of mitotic cell cycle	314	37	13.8	23.2	5.70E-08	<0.001
GO:0044428	nuclear part	1762	125	77.7	47.3	6.60E-08	<0.001
GO:0000956	nuclear-transcribed mRNA catabolic process	34	11	1.5	9.5	1.30E-07	<0.001
GO:0007067	mitosis	311	36	13.7	22.3	1.30E-07	<0.001
GO:0006402	mRNA catabolic process	36	11	1.6	9.4	2.50E-07	<0.001
GO:0003723	RNA binding	1019	80	44.9	35.1	4.00E-07	0.002
GO:0022403	cell cycle phase	549	51	24.2	26.8	4.70E-07	0.002
GO:0006401	RNA catabolic process	39	11	1.7	9.3	6.20E-07	0.002
GO:0006396	RNA processing	689	59	30.4	28.6	9.20E-07	0.002
GO:0008380	RNA splicing	380	39	16.7	22.3	9.40E-07	0.002
GO:0043285	biopolymer catabolic process	905	72	39.9	32.1	9.50E-07	0.002
GO:0006397	mRNA processing	464	44	20.4	23.6	1.70E-06	0.002
GO:0022402	cell cycle process	655	56	28.9	27.1	1.80E-06	0.002
GO:0009057	macromolecule catabolic process	1025	78	45.2	32.8	1.80E-06	0.002
GO:0043283	biopolymer metabolic process	7835	414	345.3	68.7	2.30E-06	0.002
GO:0043170	macromolecule metabolic process	8106	426	357.2	68.8	2.60E-06	0.004
GO:0000279	M phase	432	41	19	22	3.70E-06	0.006
GO:0051726	regulation of cell cycle	463	43	20.4	22.6	3.70E-06	0.006
GO:0051301	cell division	405	39	17.8	21.2	4.40E-06	0.006
GO:0044260	cellular macromolecule metabolic process	7947	417	350.2	66.8	4.50E-06	0.006
GO:0008026	ATP-dependent helicase activity	131	19	5.8	13.2	4.80E-06	0.006
GO:0070035	purine NTP-dependent helicase activity	131	19	5.8	13.2	4.80E-06	0.006
GO:0044265	cellular macromolecule catabolic process	966	73	42.6	30.4	5.10E-06	0.008
GO:0034960	cellular biopolymer metabolic process	7783	409	343	66	5.20E-06	0.008

Table 1. Gene Ontology analysis of transcripts of top 1000 ranked probe sets for stabilization by IL-3.
