# Supplementary figures and images for: IL-3 and Oncogenic Abl Regulate the Myeloblast Transcriptome by Altering mRNA Stability
Source: PLoS One. 2009 Oct 15;4(10):e7469. doi: 10.1371/journal.pone.0007469 (PMC2758590; doi:10.1371/journal.pone.0007469)

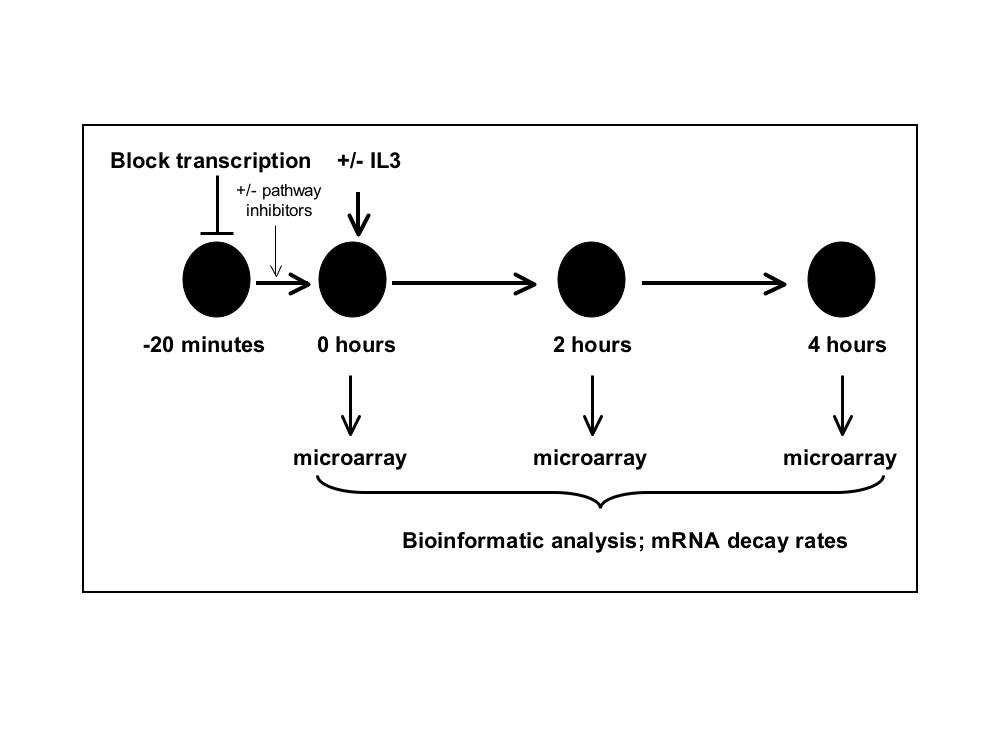

Supplement: Figure S1 — Schematic of data collection for global transcript decay analysis. Following actinomycin D addition, cells were cultured in IL-3 replete or deficient medium starting at time 0. In followup Northern Blot experiments, pathway inhibitors were added 15–20 minutes before IL-3 or vehicle control was added. (0.07 MB TIF) [file pone.0007469.s006.tif]

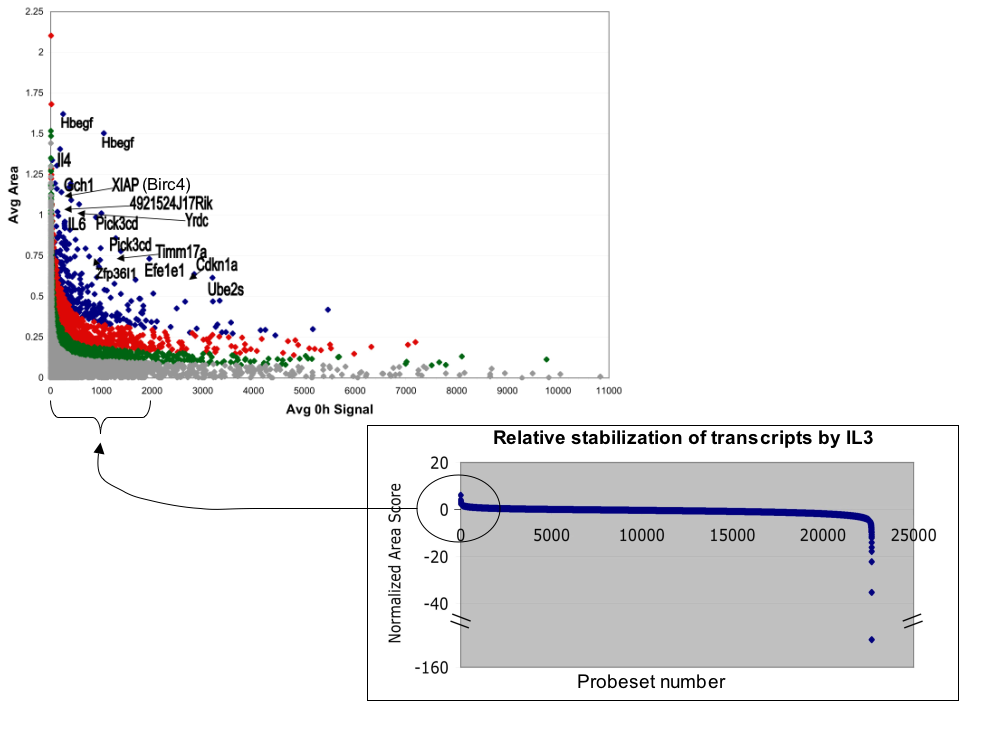

Supplement: Figure S2 — Top: Stability plots of myeloblast transcript probe sets determined using kinetic microarrays. In these plots, the garea value (y-axis) is plotted against the intensity of the probe signal at time 0 (x-axis. Increased stability correlates with positions farthest from the origin. The highest ranked 250, 1000, or 2000 probe sets based on gscore as well as all probe sets with positive garea values (5290 total) are shown and highlighted by color. The plots of the top 250 Blue), 1000 (red), and 2000 (green) and all positive probe sets (grey) in terms of the difference between their decay curve in IL-3 -replete and -deficient medium are overlaid. The top 15 ranking probe sets are labeled with their corresponding gene names. The identities and ranking of all 22,690 probe sets can be found in Supplemental Table 1. The top 1000 probesets account for 53% of the total cumulative garea attributable to IL3-stabilization. Bottom: The difference between transcript half-lifes in IL3 and in IL3-deficient conditions (normalized area score) is plotted for 22690 probe sets arranged from most stabilized by IL-3 (positive values) to least stabilized by IL-3 (negative values; less stable in IL-3). The broken axis facilitates notation of the last probeset (rank 22690) with a value of −148. The region corresponding to the indicated probesets on the top graph is circled. (0.17 MB TIF) [file pone.0007469.s007.tif]
